# Comparative analysis of the trends in laryngeal cancer disease burden between China and the world from 1990 to 2021

**DOI:** 10.1097/MD.0000000000051127

**Published:** 2026-10-16

**Authors:** Hongming Liao, Hongyong Wang

**Affiliations:** a Department of Otolaryngology Head and Neck Surgery, The First People’s Hospital of Tianmen City (Tianmen Hospital Affiliated to Wuhan University of Science and Technology), Tianmen, Hubei, China.

**Keywords:** China, disease burden, global, Global Burden of Disease, laryngeal cancer

## Abstract

Laryngeal cancer remains an important public health concern, with substantial variation in disease burden across regions, age groups, and sexes. This study aimed to characterize temporal trends in the burden of laryngeal cancer in China from 1990 to 2021 and to compare age- and sex-specific patterns of incidence, prevalence, mortality, and disability-adjusted life years (DALYs) with global trends, using data from the Global Burden of Disease 1990 to 2021 study. Joinpoint regression was applied to calculate average annual percentage changes and corresponding 95% confidence intervals to quantify long-term trends. During the study period, China’s age-standardized prevalence rate declined slightly from 1.82 to 1.789 per 100,000, while the global prevalence rate decreased more markedly from 3.072 to 2.293 per 100,000. In contrast, the age-standardized incidence rate in China increased from 7.83 to 9.864 per 100,000, whereas a global decline was observed from 15.274 to 12.558 per 100,000. Age-standardized mortality and DALY rates in China decreased substantially, from 40.372 to 22.726 per 100,000 and from 1.59 to 0.937 per 100,000, respectively, paralleling but remaining lower than global reductions. The average annual percentage changes in China for DALYs, mortality, incidence, and prevalence were −1.89, −1.73, −0.10, and 0.74, respectively, compared with global values of −1.60, −1.47, −0.95, and −0.65. Notably, males consistently exhibited a significantly higher burden than females across all indicators. These findings highlight divergent trends between China and the global population, particularly the rising incidence in China despite declining mortality and DALYs, underscoring the need to strengthen early screening and prevention strategies for laryngeal cancer, especially among high-risk male populations.

## 1. Introduction

Cancer poses a significant public health challenge worldwide, with substantial impacts on both incidence and mortality rates.^[1]^ Understanding the temporal trends in cancer burden is crucial for devising effective prevention and management strategies. Between 1990 and 2021, the burden of cancer has fluctuated across different regions,^[2]^ influenced by factors such as genetics, environmental exposures, and socio-economic conditions.Despite being largely preventable, laryngeal cancer remains a serious global public health concern due to its early onset, high mortality rate, and significant disability, particularly in many low-income countries. In China, the rapid development of the healthcare system and socio-economic changes present a unique context for studying these trends. Historically, laryngeal cancer incidence in China has been shaped by high tobacco consumption and industrial pollution.^[3]^However, recent advancements in public health infrastructure and cancer prevention programs may have shifted the burden of this disease. Comparing these trends with global data can shed light on the effectiveness of interventions and reveal regional disparities.

The Global Burden of Disease (GBD) study provides a comprehensive dataset to analyze these trends and compare the burden of laryngeal cancer across different countries.^[4,5]^ This research seeks to examine the temporal trends in laryngeal cancer in China and contrast them with global patterns.By analyzing age- and sex-specific incidence, prevalence, mortality, and disability-adjusted life years (DALYs), this study will offer an in-depth understanding of how the burden of laryngeal cancer has evolved and pinpoint areas for targeted intervention. Joinpoint regression analysis will be used to investigate these temporal trends and evaluate changes in the burden over the past thirty years, offering valuable insights for policymakers to assess the overall burden of laryngeal cancer in China and aid in the development of targeted prevention strategies and the equitable distribution of public health resources.

## 2. Materials and methods

### 2.1. Methods

This study utilized open data from the GBD study covering the period from 1990 to 2021 to analyze the burden of laryngeal cancer both in China and globally. Key metrics of interest included incidence, prevalence, mortality, and DALYs, This study was approved by the Ethics Committee of Tianmen First People’s Hospital (Approval No. 20240301).

### 2.2. Data sources and metrics

This study was approved by the Ethics Committee of Tianmen First People’s Hospital (approval number: 20240301), and all participants provided written informed consent prior to study initiation. The research was conducted in accordance with the Declaration of Helsinki and relevant local regulations. Data were obtained from the Global Health Data Exchange (GHDx) and its associated analytical tools (http://ghdx.healthdata.org/gbd-results-tool),^[6–8]^ which provide comprehensive and standardized information on disease burden indicators.

The following metrics were analyzed:

Incidence Rate: The number of new laryngeal cancer cases per 100,000 people.
Prevalence Rate: The total number of laryngeal cancer cases per 100,000 people at a given time.
Mortality Rate: The number of deaths due to laryngeal cancer per 100,000 people.
DALYs: A composite measure that includes years of life lost due to premature mortality and years lived with disability due to laryngeal cancer.

Data modeling utilized standardized tools to estimate these metrics by age, sex, location, and year. The GBD tools provided a robust dataset for assessing laryngeal cancer burden over the specified period.

### 2.3. Statistical analysis

To examine trends in laryngeal cancer burden, Joinpoint regression analysis was employed. This technique evaluated the temporal changes in the metrics of interest, including age-standardized rates (age-standardized incidence rate [ASIR], age-standardized prevalence rate [ASPR], age-standardized mortality rate [ASMR], and age-standardized DALY rate [ASDR]) and crude rates across different age groups.

Joinpoint regression was performed using Joinpoint software (National Cancer Institute, Rockville) to compute the average annual percentage change (AAPC) the AAPC and corresponding 95% confidence intervals (95%CI) for each metric. The regression model applied to log-transformed age-standardized indicators followed the equation ln(y)=α+βx+ε,where y represents the age-standardized metric and × denotes the calendar year. AAPC was calculated as 100 × (exp(β)-1), with 95%CI derived from the model. Trends were classified as increasing if the AAPC and 95% CI were >0, decreasing if <0, and stable if the CI included 0.

Statistical analysis and data visualization were conducted using R statistical software (version 4.2.2) and Joinpoint software (version 4.9.1.0). A *P*-value of <.05 was considered statistically significant.

This approach allows for a detailed examination of how the burden of laryngeal cancer has evolved over time and provides insights into regional disparities and the effectiveness of public health interventions.

## 3. Results

### 3.1. Overview of the laryngeal cancer burden in China and around the world

#### 3.1.1. Laryngeal cancer incidence rates in China and worldwide

In China, the number of laryngeal cancer cases surged from 15,434 (95%CI:12,624–18,174) in 1990 to 38,905 (95%CI:30,370–49,486) in 2021, reflecting a cumulative rise of 152.07%. On a global scale, the incidence increased from 125,175 cases (95%CI:118,981–131,639) in 1990 to 200,883 cases (95%CI:186,941–216,098) in 2021, a cumulative growth of 60.48%. While the global ASIR declined from 3.072 per 100,000 population (95%CI: 14.538–16.064) in 1990 to 2.293 per 100,000 (95%CI:11.757–13.493) in 2021, the ASIR in China declined from 1.82 per 100,000 (95%CI:6.466–9.125) in 1990 to 1.789 per 100,000 (95%CI:7.811–12.348) in 2021. The AAPC in incidence rates from 1990 to 2021 was −0.1% (95%CI: −0.16–−0.05) in China, compared to a global decrease of −0.95% (95%CI: −0.97–−0.93) during the same period (Table 1).

**Table 1:** Cases and age-standardized rates of incidence, prevalence, mortality, and disability, along with annual average percentage changes (AAPC) for laryngeal cancer in China and globally by age group for 1990 and 2021.

Location	Measure	1990		2021		1990–2021 AAPC
		All-ages cases	Age-standardized rates per 100,000 people	All-ages cases	Age-standardized rates per 100,000 people	
		n (95% CI)	n (95% CI)	n (95% CI)	n (95% CI)	n (95% CI)
China	DALYs (Disability-Adjusted Life Years)	362,503 (295,796–428,646)	40.371 (33.134–47.599)	493,848 (382,572–626,010)	22.726 (17.67–28.653)	−1.89 (−1.95–1.85)
	Deaths	12,870 (10,565–15,143)	1.59 (1.319–1.86)	19,799 (15,580–25,023)	0.937 (0.743–1.174)	−1.73 (−1.79–1.69)
	Incidence	15,434 (12,624–18,174)	1.82 (1.503–2.133)	38,905 (30,370–49,486)	1.789 (1.405–2.263)	−0.1 (−0.16–0.05)
	Prevalence	69,309 (56,887–81,340)	7.83 (6.466–9.125)	217,849 (171,469–273,851)	9.864 (7.811−12.348)	0.74 (0.7–0.78)
Global	DALYs (Disability-Adjusted Life Years)	2,475,842 (–2,636,993)	59.303 (55.472–63.098)	3,143,309 (–3,383,514)	35.803 (33.294–38.538)	−1.6 (−1.63–1.58)
	Deaths	85,790 (80,409–91,208)	2.145 (2.012–2.28)	117,252 (109,355–125,952)	1.35 (1.259–1.449)	−1.47 (−1.49–1.44)
	Incidence	125,175 (118,981–131,639)	3.072 (2.92–3.228)	200,883 (186,941–216,098)	2.293 (2.133–2.466)	−0.95 (−0.97–0.93)
	Prevalence	628,532 (598,814–660,379)	15.274 (14.538–16.064)	1,103,684 (–1,186,560)	12.558 (11.757–13.493)	−0.65 (−0.67–0.63)

#### 3.1.2. Prevalence of laryngeal cancer in China and worldwide

In terms of prevalence, the number of laryngeal cancer cases in China increased from 69,309 (95%CI:56,887–81,340) in 1990 to 217,849 (95%CI:171,469–273,851) in 2021, marking a cumulative rise of 214.32%. Conversely, the global prevalence grew from 628,532 cases (95%CI: 598,814–660,379) in 1990 to 1103,684 cases (95%CI:1033,145–1186,560) in 2021, representing a cumulative increase of 75.60%. The global average prevalence rate declined from 15.274 per 100,000 population (95%CI:2.92–3.228) in 1990 to 12.558 per 100,000 population (95%CI:2.133–2.466) in 2021. In China, the average prevalence rate increased from 7.83 per 100,000 population (95%CI:1.503–2.133) in 1990 to 9.864 per 100,000 population (95%CI:1.405–2.263) in 2021. Over the period from 1990 to 2021, the AAPC in global prevalence rates declined by 0.65% (95%CI: −0.67–−0.63), whereas the AAPC for prevalence rates in China increased by 0.74% (95% CI: 0.7–0.78) (Table 1).

#### 3.1.3. Laryngeal cancer mortality rates in China and globally

In China, the number of deaths from laryngeal cancer increased from 12,870 (95%CI:10,565–15,143) in 1990 to 19,799 (95%CI:15,580–25,023) in 2021, a rise of 53.84% over the period. Conversely, globally, laryngeal cancer mortality rose from 85,790 deaths (95%CI:80,409–91,208) in 1990 to 117,252 deaths (95%CI:109,355–125,952) in 2021, marking a 36.67% increase. In China, the ASMR decreased from 1.59 per 100,000 population (95%CI:1.319–1.86) in 1990 to 0.937 per 100,000 population (95%CI:0.743–1.174) in 2021. Globally, the ASMR dropped from 2.145 per 100,000 population (95%CI:55.472–63.098) in 1990 to 1.35 per 100,000 population (95%CI:33.294–38.538) in 2021. Between 1990 and 2021, the AAPC in global mortality rates decreased by 1.47% (95%CI: −1.49–−1.44), whereas in China, it decreased by 1.73% (95% CI: −1.79–−1.69) (Table 1).

#### 3.1.4. DALYs for laryngeal cancer in China and globally

On a global scale, DALYs attributed to laryngeal cancer increased from 2475,842 (95%CI:2313,814–2636,993) in 1990 to 3143,309 (95%CI:2922,792–3383,514) in 2021, marking a 26.96% rise. In China, DALYs for laryngeal cancer rose from 362,503 (95%CI:295,796–428,646) in 1990 to 493,848 (95%CI:382,572–626,010) in 2021, showing a 36.23% increase. The global age-standardized disability rate (ASDR) declined from 59.303 per 100,000 individuals (95%CI:2.012–2.28) in 1990 to 35.803 per 100,000 individuals (95%CI:1.259–1.449) in 2021. Similarly, in China, the ASDR decreased from 40.371 per 100,000 individuals (95% CI: 1.319–1.86) in 1990 to 22.726 per 100,000 individuals (95%CI:0.743–1.174) in 2021. From 1990 to 2021, the AAPC in global DALYs decreased by 1.6% (95%CI: −1.63–−1.58), while in China, it saw a reduction of 1.89% (95%CI: −1.95–−1.85) (Table 1).

### 3.2. Regression analysis of laryngeal cancer burden in China and globally using joinpoint

The figures presented in this paper illustrate the Joinpoint regression analysis of the ASIR, ASPR, ASMR, and DALYs associated with laryngeal cancer in China and globally from 1990 to 2021. In China, the annual percentage change (APC) in ASIR showed an initial decline (ASIR:1990–1999, APC = −1.16, *P* < .05; 1999–2007, APC = −0.32, *P* < .05), which was followed by an increase (ASIR: 2007–2011, APC = 1.97, *P* < .05; 2011–2021, APC = 0.22, *P* > .05). Similarly, the APC for ASPR first decreased (ASPR:1990–1998, APC = -0.45, *P* < .05; 1998–2003, APC = −0.1, *P* > .05) before rising (ASPR:2003–2007, APC = 1.66, *P* < .05; 2007–2012, APC = 2.40, *P* < .05; 2012–2016, APC = 0.5, *P* < .05; 2016–2021, APC = 1.32, *P* < .05). On the global scale, there was a modest increase in APC for ASIR and ASPR around 1995, after which both steadily declined until 2021. Throughout this period, significant reductions were observed in ASMR and DALYs for laryngeal cancer in both China and globally (Fig. 1 and Fig. 2).

**Figure 1.**
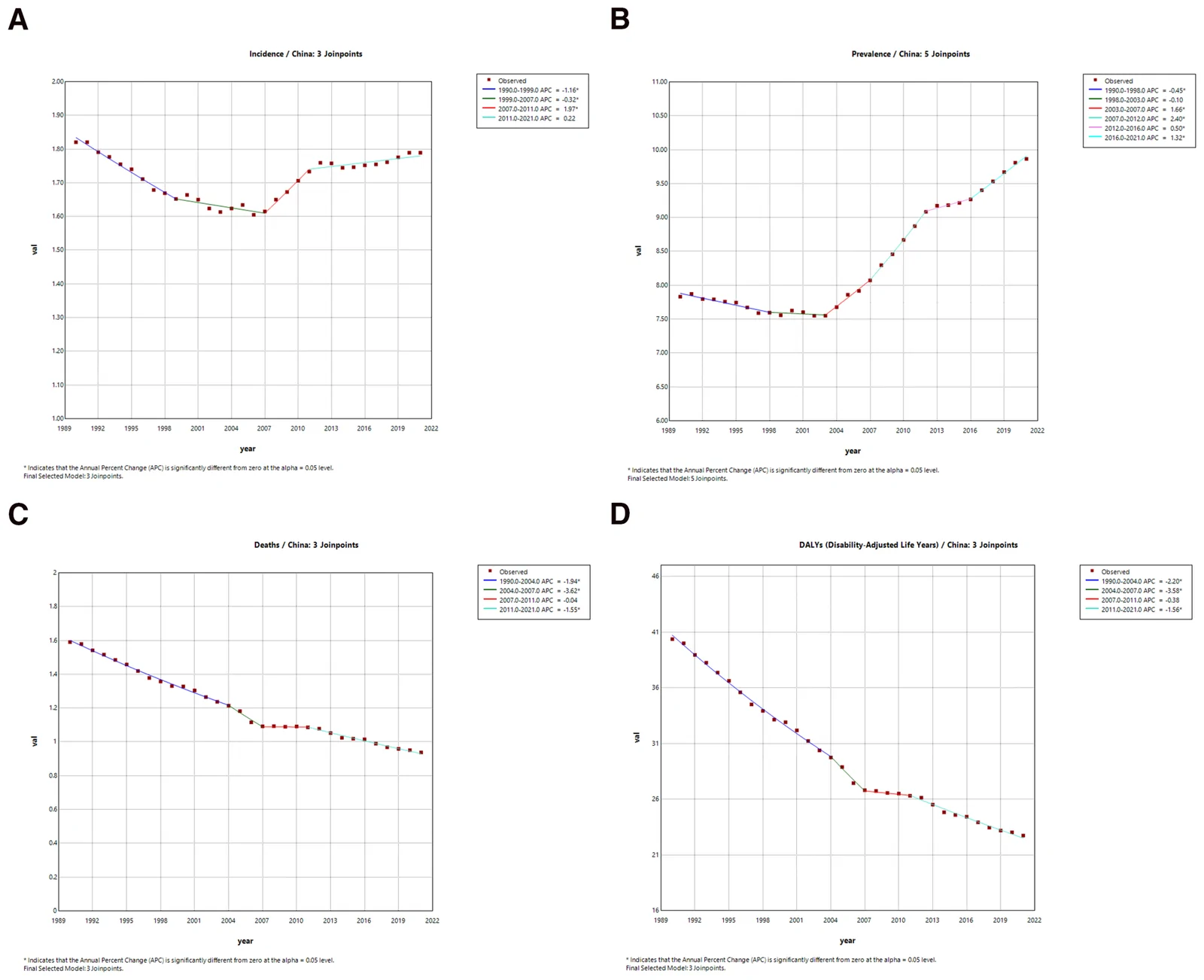
Compares the age-standardized rates of incidence, prevalence, mortality, and DALYs for laryngeal cancer across different age groups in China from 1990 to 2021. (A) Age-Standardized Incidence Rate (ASIR), (B) Age-Standardized Prevalence Rate (ASPR), (C) Age-Standardized Mortality Rate (ASMR), (D) Disability-Adjusted Life Years (DALYs).

**Figure 2.**
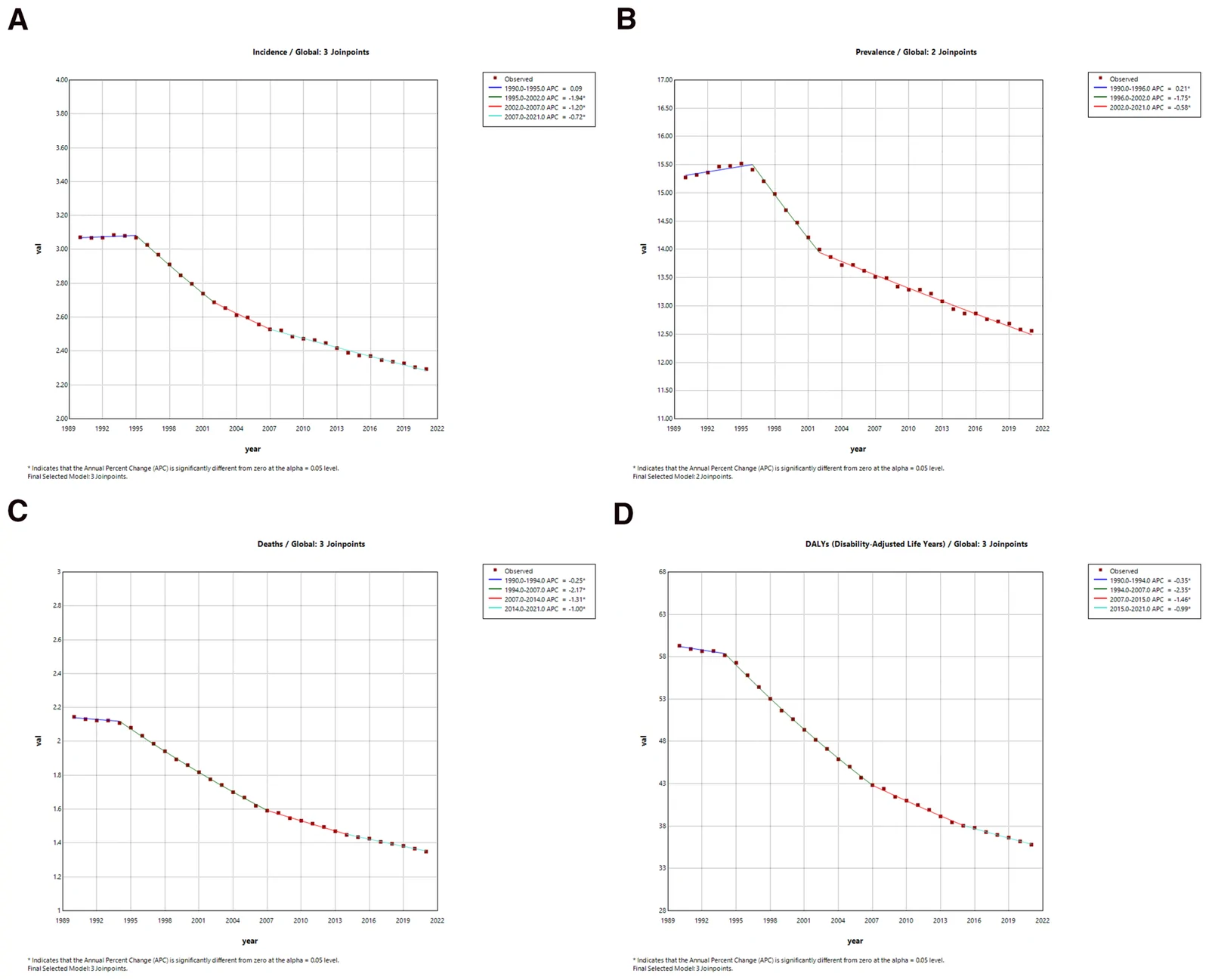
Compares the age-standardized rates of incidence, prevalence, mortality, and DALYs for laryngeal cancer across different age groups globally from 1990 to 2021. (A) Age-Standardized Incidence Rate (ASIR), (B) Age-Standardized Prevalence Rate (ASPR), (C) Age-Standardized Mortality Rate (ASMR), (D) Disability-Adjusted Life Years (DALYs).

### 3.3. Trends in the disease burden of laryngeal cancer in China and globally

Between 1990 and 2021, DALYs for laryngeal cancer have exhibited a gradual decline both in China and worldwide, with a notable overall decreasing trend. In contrast, ASPR for laryngeal cancer has been rising in China, while the global ASPR showed a period of slow increase followed by a decline, ultimately trending downwards. Furthermore, ASIR and ASMR for laryngeal cancer have displayed relatively stable trends in both China and globally (Fig. 3).

**Figure 3.**
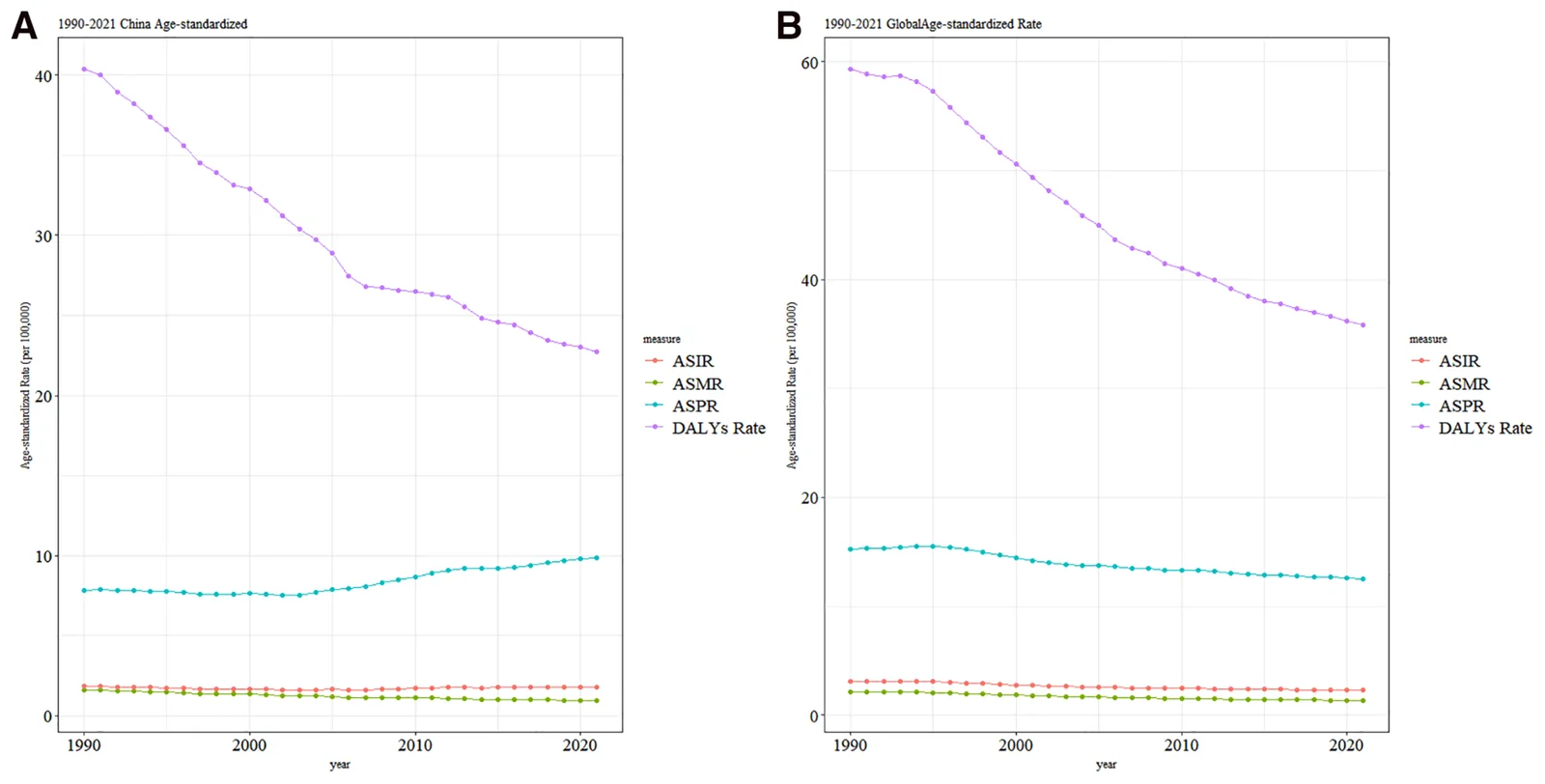
Compares the trends in ASIR, ASMR, ASPR, and DALYs for laryngeal cancer in China and globally from 1990 to 2021. (A) Age-Standardized Incidence Rate (ASIR), (B) Age-Standardized Prevalence Rate (ASPR), (C) Age-Standardized Mortality Rate (ASMR), (D) Disability-Adjusted Life Years (DALYs).

### 3.4. Laryngeal cancer burden across different age groups in China for the years 1990 and 2021

Figure 4 illustrates the incidence, prevalence, mortality rates, and DALYs of laryngeal cancer by age group in China for 1990 and 2021, including the corresponding crude rates. The data show an increase in incidence rates after age 50, with the highest cases in the 65 to 69 age group. Prevalence rates are highest in the 50 to 80 age range, rising and then falling with age. Mortality rates peak in the 65 to 69 and 70 to 74 age groups. DALYs peaked in the 55 to 59 age group in 1990 and shifted to the 65 to 69 age group in 2021.

**Figure 4.**
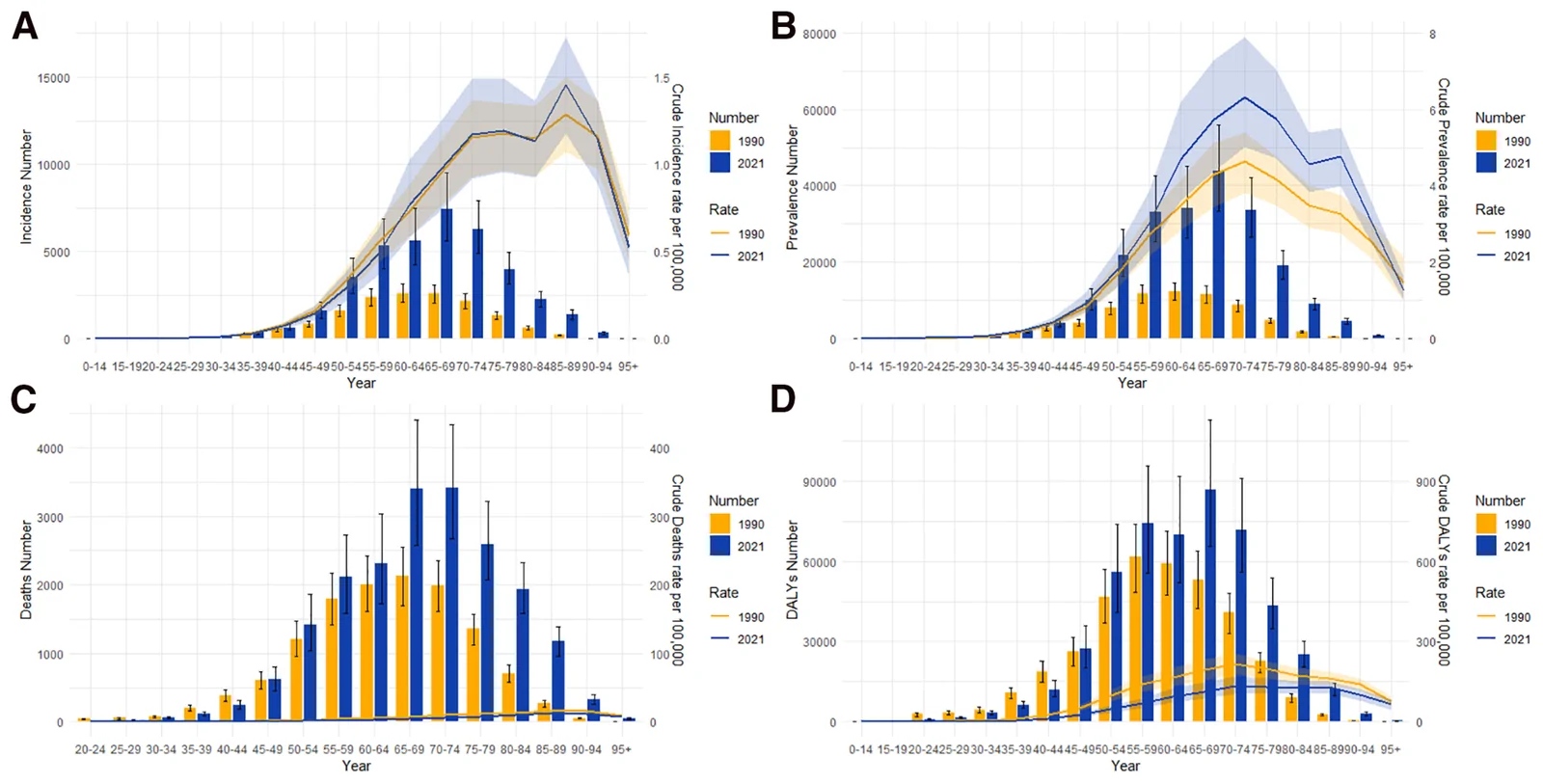
Compares the incidence, prevalence, mortality rates, and disability-adjusted life years (DALYs) by age group in China from 1990 to 2021, along with crude rates. (A) Incidence and cases; (B) Prevalence and cases; (C) Mortality and cases; (D) Disability-adjusted life years (DALYs). Bar graphs represent counts, while lines depict crude rates.

### 3.5. Age-specific gender differences in laryngeal cancer burden in China

Figures 5 and 6 present the incidence rates, prevalence rates, mortality rates, and DALYs for laryngeal cancer by age group in China for 1990 and 2021, disaggregated by gender. The data indicate that for both years, women experienced the highest incidence in the 65 to 69 age group, while men peaked in the 60 to 64 age group, with men having significantly more cases than women. For prevalence in 1990, the top age groups for women were 65 to 69, 60 to 64, and 70 to 74, while for men they were 60 to 64, 50 to 59, and 65 to 69, which aligns with the 2021 trends. Regarding mortality, men consistently had higher death rates than women across all age groups, with peaks at 65 to 69 for men and 70 to 74 for women. DALYs followed a similar pattern, showing higher rates for men across all age groups. In 1990, the highest DALYs for women were in the 65 to 69 age group, and for men, in the 55 to 59 age group, reflecting the trends seen in 2021.

**Figure 5.**
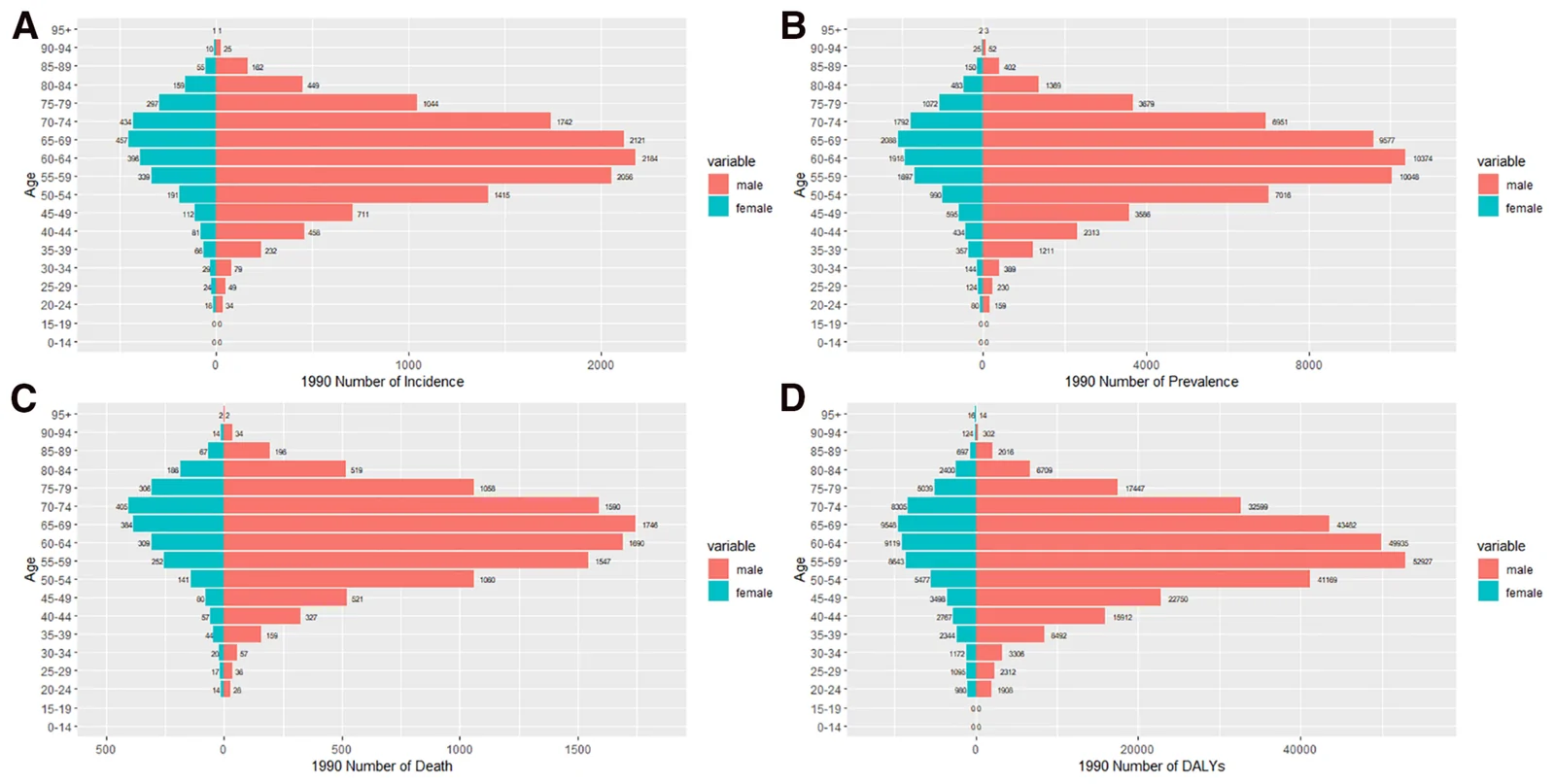
Comparison of laryngeal cancer incidence, prevalence, mortality, and disability-adjusted life years (DALYs) by age group in China for 1990. (A) Incidence count; (B) Prevalence count; (C) Mortality count; (D) Disability-adjusted life years (DALYs) count.

**Figure 6.**
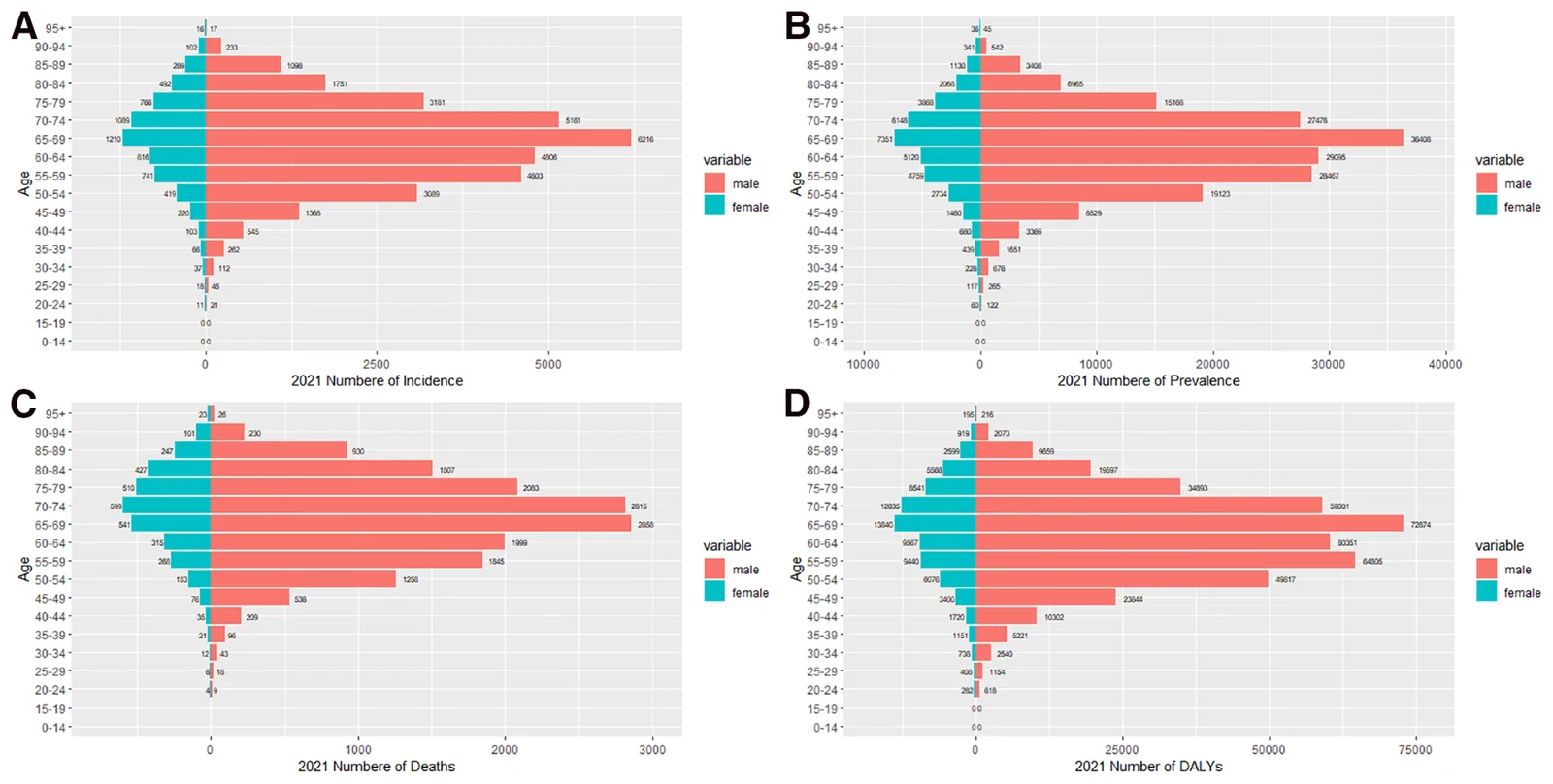
Comparison of laryngeal cancer incidence, prevalence, mortality, and Disability-Adjusted Life Years (DALYs) by age group in China for 2021. (A) Incidence count; (B) Prevalence count; (C) Mortality count; (D) Disability-adjusted life years (DALYs) count.

Figure 7 illustrates the laryngeal cancer burden and age-standardized rates for men and women in China from 1990 to 2021. For men, ASIR, ASPR, ASMR, and DALYs rise with age. Conversely, these rates increase more gradually with age for women, reflecting their lower overall case numbers.

**Figure 7.**
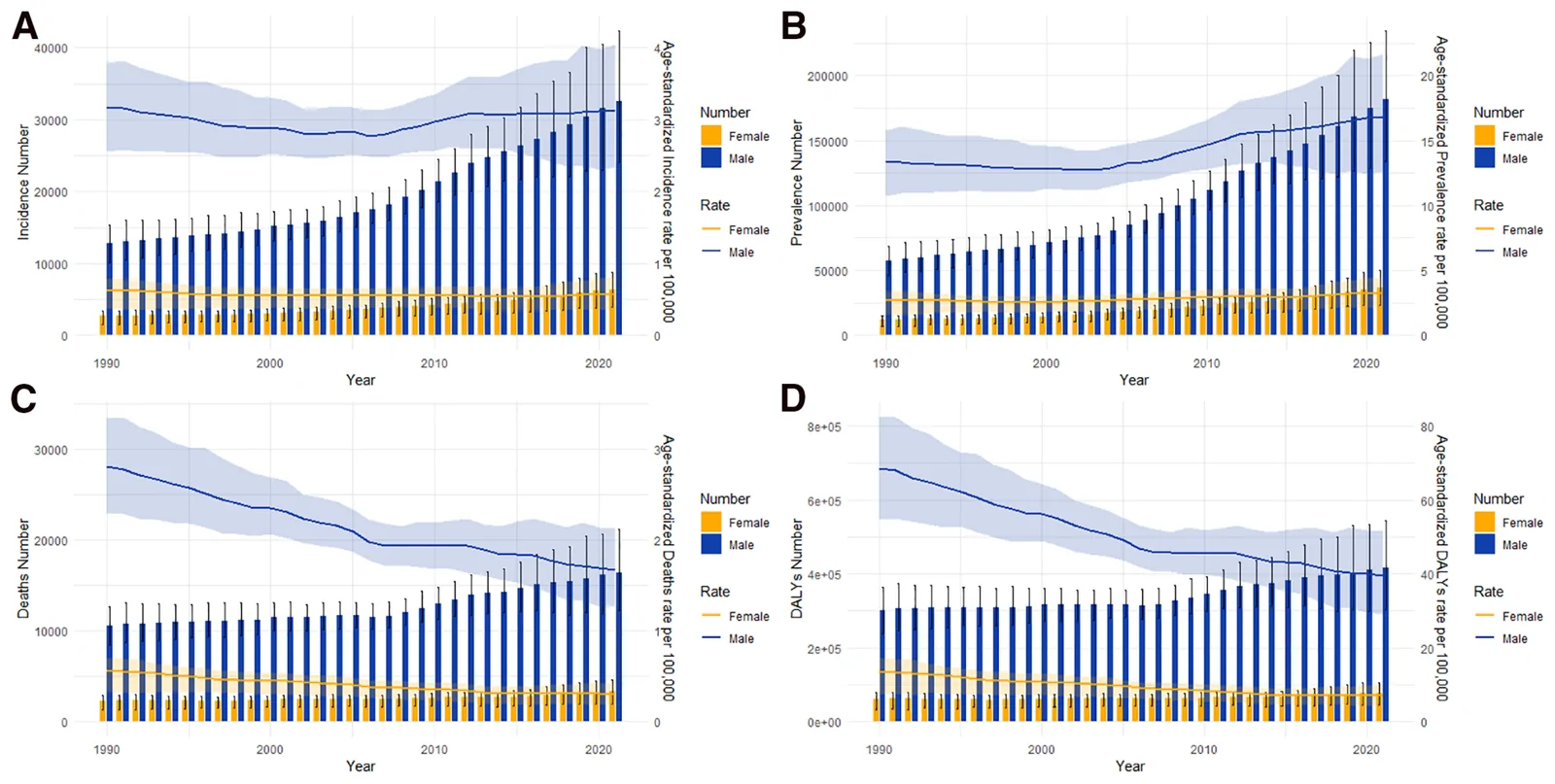
Comparison of laryngeal cancer incidence, prevalence, mortality, and Disability-Adjusted Life Years (DALYs) by gender and age-standardized rates in China from 1990 to 2021. (A) Incidence rates and counts; (B) Prevalence rates and counts; (C) Mortality rates and counts; (D) Disability-Adjusted Life Years (DALYs); Bar graphs represent counts; line graphs represent crude rates.

## 4. Discussion

Analysis based on the most recent 2021 GBD data highlights significant trends in the evolution of ASIR, ASPR, ASMR, and DALYs for laryngeal cancer. From 1990 to 2021, the global incidence rate surged by 60.48%, and prevalence increased by 75.60%. During the same period, global mortality due to laryngeal cancer rose by 36.67%, while DALYs grew by 26.96%. Notably, the increases in ASIR and ASPR were markedly greater compared to the changes in ASMR and DALYs. These trends may be driven by various factors, including shifts in environmental and lifestyle factors, advancements in public health systems, an aging population, and evolving definitions and classifications of the disease. Understanding these trends is crucial for the strategic allocation and planning of health resources. As the global population continues to grow and age, the demand for specialized laryngeal cancer care is anticipated to rise, intensifying the overall burden.

This study reveals that cancer incidence is significantly higher in men compared to women, primarily due to higher rates of tobacco use and excessive alcohol consumption among men. Historically, men have smoked at much higher rates than women,^[9]^ and while smoking rates among women have increased in some regions, men’s overall exposure remains greater. Additionally, men tend to consume alcohol more frequently and in larger quantities, which further elevates their risk of laryngeal cancer.^[10]^Lewin et al reported that the relative risk of head and neck cancers in current smokers is 6.5 times higher than in nonsmokers, while the relative risk associated with alcohol consumption is 5.5%,Their findings suggest a nearly multiplicative effect of smoking and drinking on cancer risk.^[11,12]^Occupational exposure is another significant factor.^[13,14]^ Jobs that involve contact with carcinogens such as asbestos, wood dust, and industrial chemicals are more prevalent among men, thus increasing their cancer risk. Sex hormones, such as testosterone and estrogen, may also influence cancer risk, with testosterone potentially increasing susceptibility, whereas estrogen might offer some protective effects.^[15–17]^ Genetic predisposition further contributes, with men possibly having a higher genetic risk for laryngeal cancer due to differences in genetic mutations and repair mechanisms.^[18]^ Behavioral and social factors also play a role; men are generally less likely to seek early medical care or screening, resulting in later-stage diagnoses. Lastly, historical and cultural factors, including historically higher rates of tobacco and alcohol use among men, continue to impact current cancer statistics.

Data from the 2021 GBD study shows that laryngeal cancer incidence was highest in men within the age groups 60 to 64, 50 to 59, and 65 to 69, while for women, it peaked in the 65 to 69, 60 to 64, and 70 to 74 age brackets. These patterns have significant implications for public health planning and resource allocation. The high incidence of laryngeal cancer in older age groups highlights the substantial burden of this disease on the aging population.^[19,20]^ In men, the peak incidence in the 60 to 64 age range suggests that this period represents the cumulative effect of risk factors such as tobacco and alcohol use, which aligns with the long latency period of laryngeal cancer, where extended exposure contributes to the disease’s onset. The overlap in age ranges for both genders, particularly the 60 to 64 and 65 to 69 groups, points to shared risk factors and possible delays in diagnosis or treatment. The later onset observed in women, especially in the 70 to 74 age group, may indicate different patterns of risk exposure or a slower disease progression compared to men. This discrepancy suggests that women may encounter distinct challenges in disease progression, including variations in preventive measures and diagnostic practices.

Despite the thoroughness of this study, several limitations must be recognized. Data constraints, including inconsistencies in reporting practices and the availability of comprehensive cancer registries, can affect the accuracy and completeness of the analyzed trends.^[21,22]^ Additionally, regional differences in healthcare access and quality may lead to variations in the data. Future research should aim to overcome these limitations by employing more precise data sources and investigating regional disparities in greater depth. Ongoing surveillance and analysis of emerging trends are vital for adapting public health strategies to effectively reduce the burden of laryngeal cancer. Longitudinal studies that incorporate recent data and assess the impact of new health interventions will offer valuable insights into the evolving trends and guide future efforts.

## 5. Conclusion

In summary, this study highlights important trends in the burden of laryngeal cancer, showcasing both advancements and ongoing issues. Persistent research and focused public health strategies are essential for effectively addressing and reducing the impact of laryngeal cancer worldwide, including in specific regions such as China.

## Acknowledgments

The authors declare that there are no conflicts of interest regarding the publication of this paper

## Author contributions

**Writing – original draft:** Hongming Liao.

**Writing – review & editing:** Hongyong Wang.
